# A High Soluble‐Fibre Allele in Wheat Encodes a Defective Cell Wall Peroxidase Responsible for Dimerization of Ferulate Moieties on Arabinoxylan

**DOI:** 10.1111/pbi.70527

**Published:** 2026-01-03

**Authors:** Rowan A. C. Mitchell, Ondrej Kosik, Abdul Kader Alabdullah, Anneke Prins, Maria Oszvald, Till K. Pellny, Jackie Freeman, Kirstie Halsey, Caroline A. Sparks, Alison Huttly, James Brett, Michelle Leverington‐Waite, Simon Griffiths, Peter R. Shewry, Alison Lovegrove

**Affiliations:** ^1^ Plant Sciences, Rothamsted Research Hertfordshire UK; ^2^ John Innes Centre Norwich UK; ^3^ Earlham Institute Norwich UK

**Keywords:** arabinoxylan cross‐linking, feruloyl‐arabinoxylan, peroxidase‐mediated ferulate dimerization, soluble dietary fibre, wheat endosperm cell wall

## Abstract

Increasing dietary fibre (DF) intake is an important target to improve health. An attractive strategy for this is to increase DF in wheat which is derived principally from the endosperm cell wall polysaccharide arabinoxylan (AX). The water‐extractable form of this (WE‐AX) accounts for most soluble dietary fibre (SDF), which is believed to confer particular health benefits. A region of chromosome 6B in some wheat varieties confers high SDF and here we show that the cause is an allele encoding a peroxidase family protein with a single residue change (PER1‐v) associated with high WE‐AX, compared to the more common form (PER1). Both wheat lines carrying this natural PER1‐v variant and those with an induced knockout mutation of PER1 showed reduced dimerization of endosperm ferulate consistent with a mechanism of decreased cross‐linking in the cell wall that increases WE‐AX. Transiently expressed PER1_RFP fusion protein driven by the native promoter in wheat endosperm was shown to localise to cell walls, whereas PER1‐v_RFP did not. We therefore propose that PER1‐v lacks the capacity to dimerise AX ferulate in vivo due to mis‐localisation caused by the missense single‐nucleotide polymorphism (SNP) in the PER1‐v allele, so that the SNP acts as a perfect marker. This marker can be used to identify current wheat varieties with high WE‐AX to be used by processors and by breeders to ensure future varieties have high WE‐AX to make healthier wheat‐based foods.

## 1 Introduction

Dietary fibre (DF) is an essential component of the human diet and the intake of cereal fibre is associated with a reduced risk of a range of chronic diseases such as type 2 diabetes and colorectal cancer (Gill et al. 2021; Reynolds et al. 2019). The mechanisms of action of fibre are complex and still incompletely understood. However, it has been suggested that the balance of three properties, solubility, viscosity and fermentability in the colon, is important in determining the behaviour of fibre in the GI tract (Gill et al. 2021). Hence, soluble and insoluble forms of fibre share some health benefits but also differ in others. In particular, soluble fibre may have benefits in the small intestine by reducing the rate of glucose release and absorption and is more readily fermented in the colon to produce beneficial short‐chain fatty acids (Gill et al. 2021).

Bread wheat is the most widely grown food crop globally accounting for ~20% of human food calories (www.fao.org/faostat) and is a major source of DF in the western diet. For example, in the UK, cereals account for about 40% of the daily intake of fibre (Bates et al. 2010) with about half of this being provided by bread (Lockyer and Spiro 2020). However, most types of bread and other wheat‐based foods are made from white flour, which has a lower content of DF than whole grain (about 4% dry wt. compared with over 10% dry wt.). Increasing the fibre content of white flour is therefore an attractive strategy to deliver health benefits to consumers. The major DF component in white flour is the endosperm cell wall polysaccharide arabinoxylan (AX), accounting for about half of the total DF (Shewry et al. 2023). The amounts of both total AX and WE‐AX in white flour vary between wheat genotypes, from about 1.35% to 2.75% dry wt. and 20% to 50% of the total, respectively (Gebruers et al. 2008). Several studies have found QTLs and Marker‐Trait Associations (MTAs) for DF, total AX and WE‐AX in wheat (Charmet et al. 2009; Hernández‐Espinosa et al. 2024; Ibba et al. 2021; Jiang et al. 2024; Lovegrove et al. 2020; Marcotuli et al. 2015; Nguyen et al. 2011; Quraishi et al. 2011; Yang et al. 2016; Zhan et al. 2019), but no causal genes have been experimentally demonstrated.

Possible candidate genes include those involved in the synthesis of AX. AX is a chain of β‐1,4‐linked xylopyranosyl units decorated with α‐1,3‐ and α‐1,2‐linked arabinofuranosyl units; some of the α‐1,3‐linked arabinofuranosyl are themselves 5‐O‐substituted with ester‐linked feruloyl residues. These ferulate moieties are key for functionality because they allow for radical oxidative coupling to form ferulate dimers that can cross‐link chains of AX (Burr and Fry 2009; Pellny et al. 2020). This cross‐linking is believed to bind AX more tightly into the endosperm cell wall (Saulnier et al. 2007), so decreasing the extent of cross‐linking would be expected to increase the amount of WE‐AX. Cross‐linking of ferulate moieties on AX has significance beyond wheat grain, as it is a key process in all grass cell walls, determining primary wall extensibility and biomass recalcitrance to digestion and is likely a capability that contributed to the evolutionary success of the grasses (Chandrakanth et al. 2023). It has been shown that dimerization of ferulate on AX in grass cell walls requires the action of an apoplastic peroxidase (Burr and Fry 2009) but the peroxidase gene family is large (Passardi et al. 2004), and the identity of these peroxidases has not been established for any grass cell wall.

Previous studies have identified a QTL for SDF on chromosome 6B in a Yumai34 × Valoris wheat population where the Valoris allele confers high SDF (Charmet et al. 2009; Lovegrove et al. 2020). Here, we identify the causal allele in the first report of the cause of any wheat dietary fibre QTL. We demonstrate the likely mechanism of its action and show a clear path for exploiting this finding to deliver healthier wheat foods.

## 2 Results

### 2.1 RNAseq From Endosperm of Lines From Yumai34 × Valoris Population Identifies PER1 as Candidate Causal Gene

We previously mapped significant QTLs for SDF measured by relative viscosity of grain extracts on chromosomes 1B and 6B and a more minor QTL on 1A in the Yumai34 x Valoris population of doubled‐haploid lines (DHLs) (Lovegrove et al. 2020). The relative viscosity of wheat grain extracts is mostly due to WE‐AX (Freeman et al. 2016) and genetic variation in WE‐AX amount accounts for 80% of variance in relative viscosity in this population. To identify candidate genes for the 1B and 6B QTLs, we analysed RNA‐seq from pools of 4 DHLs with contrasting genotypes at these two QTLs (but all with Yumai34 high‐SDF allele at 1A).

**FIGURE 1:**
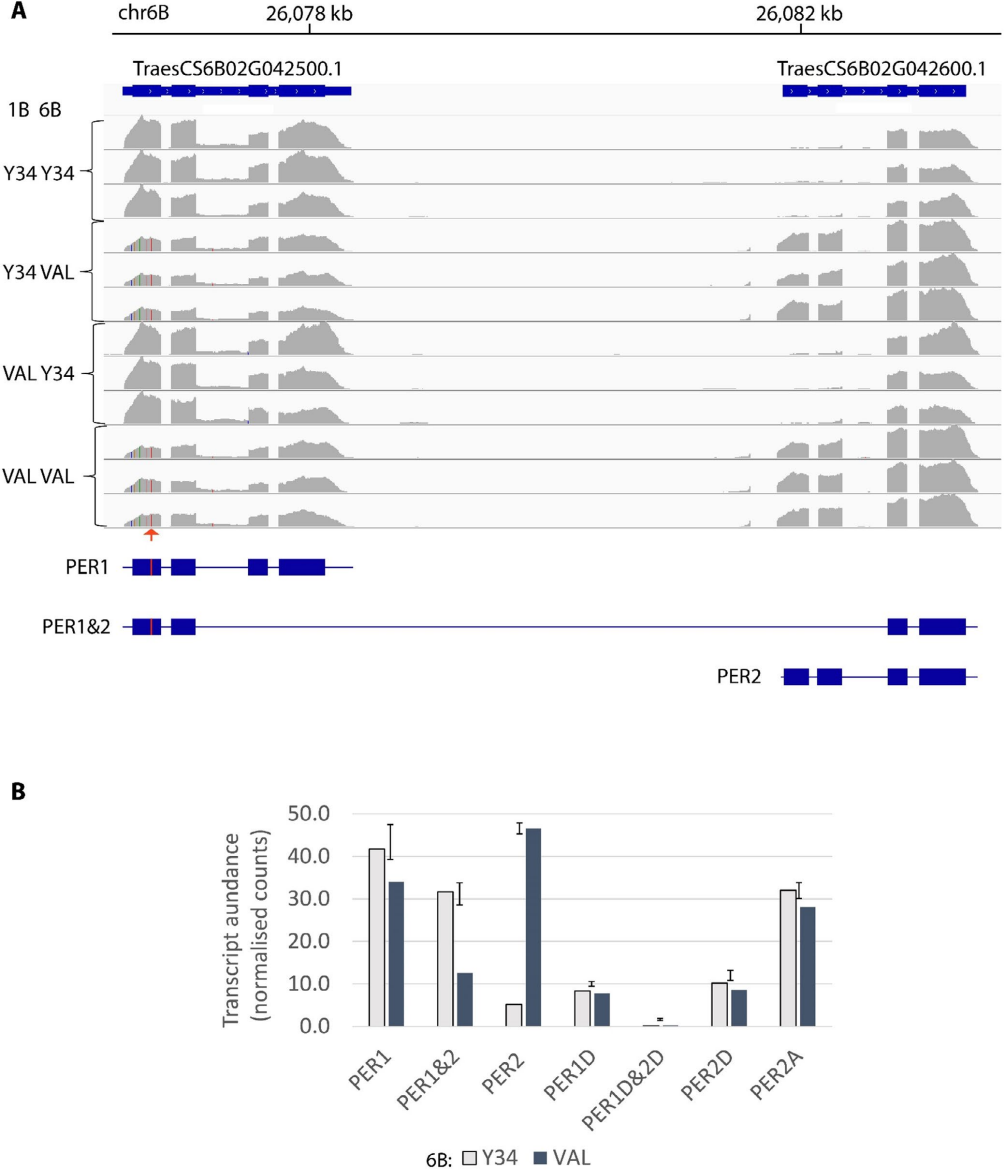
RNAseq analysis of PER transcripts from 17 dpa endosperm of DHLs in Yumai34 × Valoris mapping population. (A) Read coverage for 6B chromosome region containing TraesCS6B02G042500 and TraesCS6B02G042600 genes from 12 samples of DHLs selected to have Yumai34 (Y34) or Valoris alleles (VAL) at 1B and 6B QTLs with 3 reps of each combination. The arrow indicates the missense SNP in Valoris. Analysis of paired reads showed nearly all transcripts were one of the 3 forms depicted PER1, PER1&2 or PER2. These inferred transcripts are shown as blocks and lines where blocks represent coding exons and the missense SNP in Valoris allele is marked in red. (B) Transcript abundance of the splice variants PER1, PER1&2 or PER2 and their homeologues on sub‐genomes A and D. PER1A exons are not expressed. Bar heights and error bars are estimated means and 5% LSD for 6B alleles of each transcript from a 2‐way ANOVA of 1B and 6B allele effects. No 1B effects were significant (*p* > 0.05), so results shown group DHLs differing in 1B alleles (*n* = 6).

Transcripts for enzymes of AX synthesis typically peak around 12–18 days post anthesis (dpa) (Pellny et al. 2012), so we isolated RNA from endosperm at 17 dpa. The 95% confidence intervals [19946 488–28116185] for the 6B QTL encompass 323 protein‐coding genes annotated in wheat reference genome IWGSC Refseq 1.1; of these, 27 were expressed (average normalised counts > 0.5) and 16 were differentially expressed (FDR < 0.05) and/or had splicing differences or coding polymorphisms. From these 16, we identified neighbouring genes TraesCS6B02G042500 and TraesCS6B02G042600 annotated as peroxidases as top candidates due to their expression pattern (high in endosperm during grain fill, not expressed in non‐grain tissue) and proximity to the QTL peak. Also supportive was the classification of these peroxidases as commelinid‐specific in the universal_grass_peps database (Mitchell 2025) since feruloylated AX is confined to commelinid species. The two genes are a result of tandem duplication (sharing 85% CDS identity) and each has 4 exons. RNA‐seq mapped to these two genes (Figure 1A) shows that all abundant transcripts are made up of 4 exons but can be from either gene or from TraesCS6B02G042500 exons 1 and 2 combined with TraesCS6B02G042600 exons 3 and 4. Therefore, these two separate gene models actually represent splice variants, albeit ones sharing no exons. We named the transcripts and encoded proteins PER1 (identical to TraesCS6B02G042500.1), PER2 (identical to TraesCS6B02G042600.1) and PER1&2 for the hybrid comprised of PER1 exons 1,2 and PER2 exons 3,4. DHLs with the Valoris allele at this 6B locus showed highly significant (*p* < 0.001) differences in expression and splicing with marked decreases in PER1&2 and increases in PER2 expression (Figure 1B); the Yumai34 sequence is identical to the Chinese Spring reference, whereas Valoris PER1 has 3 synonymous SNPs and one missense SNP in exon 1 of PER1. The missense results in a Ser51Phe change in the protein encoded by the Valoris allele, which we call PER1‐v. Both PER1 and PER2 have homeologues on the A and D subgenomes, but PER1A is not expressed, and the other forms are less expressed than PER1 and PER2 (Figure 1B). We hypothesised that, despite the likely redundancy between these forms, the missense and/or lower expression of PER1‐v in Valoris is the cause of the higher SDF.

### 2.2 Wheat Lines With an Induced Mutation in PER1 Have Higher WE‐AX

For an independent test of the hypothesis that PER1 is a causal gene for 6B QTL, we used the mutagenised, exome‐sequenced population of wheat var. Cadenza (Krasileva et al. 2017) to find mutations in PER1. We identified a line carrying a premature stop codon in exon 2 of PER1, a knock‐out (KO) mutation that abolishes production of both PER1 and PER1&2 proteins. We twice backcrossed lines to Cadenza wild‐type and allowed lines to self‐fertilise for two further generations (BC2F2 lines) and identified segregants that were homozygous for the mutation and those that were homozygous for the wild‐type allele. We analysed monosaccharide composition of the WE fraction from endosperm in these lines at BC2F3 generation to estimate WE‐AX (Table 1). WE‐AX was highly significantly increased in lines carrying the PER1 KO mutation supporting the hypothesis that PER1 is the cause of 6B QTL with the high WE‐AX genotype having lower PER1 and/or PER1&2 activity.

**TABLE 1:** Monosaccharide composition in WE fraction from endosperm of Cadenza BC2F3 lines segregating for KO mutation in PER1.

		WE‐monosaccharide (mg/g dwt)			
		Xylose	Arabinose	Galactose	WE‐AX (mg/g dwt)
Means	Null	5.40	6.70	3.23	9.84
	Mutant	7.77	7.81	3.40	13.20
*F* prob.					
	Mutation	0.001	0.195	0.722	0.004

*Note:* WE‐AX content is estimated from monosaccharide composition by xylose + arabinose—0.7*galactose to correct arabinose for contribution of arabinogalactan peptide (Ordaz‐Ortiz et al. 2005). Results from a 1‐way ANOVA of mutation showing means for each group (*n* = 5, 6) and *F* probability for effect.

### 2.3 DHLs With Valoris Allele at 6B Have Lower Dimerization of Ferulate in Endosperm WE‐AX

The identification of PER1 as a candidate causal gene suggested that the mode of action for the 6B QTL could be via the dimerization of ferulate on AX, with lower dimerization associated with the Valoris PER1‐v allele. We used a method to determine bound ferulate monomer and dimer amounts released by saponification in total endosperm and water‐extractable fraction from grain. All ferulate monomer and dimers thus determined are believed to be derived from moieties ester‐linked to AX (Saulnier et al. 2007). We have previously determined dimerization of wheat endosperm ferulate in total and WE endosperm fractions, and increases were seen in both when genes responsible for the synthesis of the AX xylan backbone were downregulated (Freeman et al. 2017; Pellny et al. 2020).

Here, we observed significantly lower ferulate dimerization on WE‐AX in DHLs with the Valoris genotype (6B:H) to those with the Yumai34 genotype (6B:L) in the 6B QTL region (Table 2). Out of the five ferulate dimer (diFA) peaks detected, the ratios to FA monomer were all decreased in 6B:H DHLs and the sum of all dimers expressed as % dimerization was significantly decreased (F. prob. = 0.014) by ~30%. The effects of 1B:H were more complex, decreasing some dimers significantly but having no significant effect on overall dimerization. There were also significant effects of 1B and 6B QTLs on FA monomer amount per unit dwt endosperm with it being increased with H genotype for both QTLs (Table 2).

**TABLE 2:** Amounts of ferulate monomer (FA) and dimers (diFA) in WE fraction from endosperm of DHLs grouped according to genotype at 1B and 6B QTLs.

		Dimerisation [tot diFA/(tot diFA + FA)]	diFA/FA (w/w)					FA (μg/g dwt)
			diF8‐8AT	diF8‐8	diF8‐5	diF5‐5	diF8‐0‐4 & diF8‐5BF	
Means	1B:L 6B:L	22%	0.065	0.024	0.046	0.08	0.061	6.3
	1B:H 6B:L	19%	0.038	0.072	0.032	0.048	0.049	12.9
	1B:L 6B:H	15%	0.032	0.017	0.023	0.058	0.05	10.5
	1B:H 6B:H	14%	0.032	0.015	0.027	0.046	0.04	13.4
*F* prob.								
	1B	0.319	0.11	0.405	0.665	0.004	0.024	< 0.001
	6B	0.014	0.026	0.251	0.266	0.072	0.041	0.013
	Interaction	0.794	0.09	0.362	0.477	0.125	0.755	0.035

*Note:* Results from a 2‐way ANOVA of 1B × 6B genotype showing means for each group (*n* = 3) and *F* probability for effects.

### 2.4 Wheat Lines With KO Mutation in PER1 Have Lower Dimerization of Ferulate in Endosperm

We applied the same analysis to the BC2F3 Cadenza lines carrying the KO mutation and found similar results to those for the 6B:H genotype in Table 2; they had significantly lower ferulate dimerization in the water‐extractable fraction from endosperm and higher ferulate monomer content (Table 3). Lower dimerization supports the putative role of PER1 in promoting dimerization and higher ferulate monomer in the WE fraction is expected, as decreased dimerization results in reduced cross‐linking, making feruloyl‐AX molecules more soluble. This is consistent with a similar increase in WE‐AX in these lines (Table 1).

**TABLE 3:** Amounts of ferulate monomer (FA) and dimers (diFA) in WE fraction from endosperm of Cadenza BC2F3 lines segregating for KO mutation in PER1.

		Dimer‐isation [tot diFA/(tot diFA + FA)]	diFA/FA (w/w)					FA (μg/g dwt)
			diF8‐8AT	diF8‐8	diF8‐5	diF5‐5	diF8‐0‐4 & diF8‐5BF	
Means	Null	14.1%	0.037	0.025	0.018	0.025	0.059	12.3
	Mutant	12.2%	0.028	0.021	0.016	0.025	0.049	18.0
*F* prob.								
	Mutation	0.039	0.002	0.230	0.174	0.919	0.009	0.001

*Note:* Results from a 1‐way ANOVA of mutation showing means for each group (*n* = 5, 6) and *F* probability for effect.

We also compared ferulate content and dimerization in total endosperm in these BC2F3 lines; again dimerization was significantly decreased in mutant lines (Table 4). The magnitude of this effect is small (8%–15% decrease) but consistent; we found the same result in an earlier experiment on BCF2 generation (Table S1). We found no significant effect of the PER1 mutation on ferulate monomer content in total endosperm (Table 4; Table S1).

**TABLE 4:** Amounts of ferulate monomer (FA) and dimers (diFA) in total endosperm of Cadenza BC2F3 lines segregating for KO mutation in PER1.

		Dimer‐isation [tot diFA/(tot diFA + FA)]	diFA/FA (w/w)					FA (μg/g dwt)
			diF8‐8AT	diF8‐8	diF8‐5	diF5‐5	diF8‐0‐4 & diF8‐5BF	
Means	Null	29.8%	0.047	0.037	0.073	0.104	0.163	186.2
	Mutant	27.3%	0.042	0.037	0.063	0.089	0.146	194.5
*F* prob.								
	Mutation	0.007	0.003	0.474	0.000	0.039	0.015	0.528

*Note:* Results from a 1‐way ANOVA of mutation showing means for each group (*n* = 5, 6) and *F* probability for effect.

### 2.5 PER1_RFP Fusion Protein Transiently Expressed in Wheat Endosperm Localises Partially to Cell Wall but PER1‐v_RFP Does Not

**FIGURE 2:**
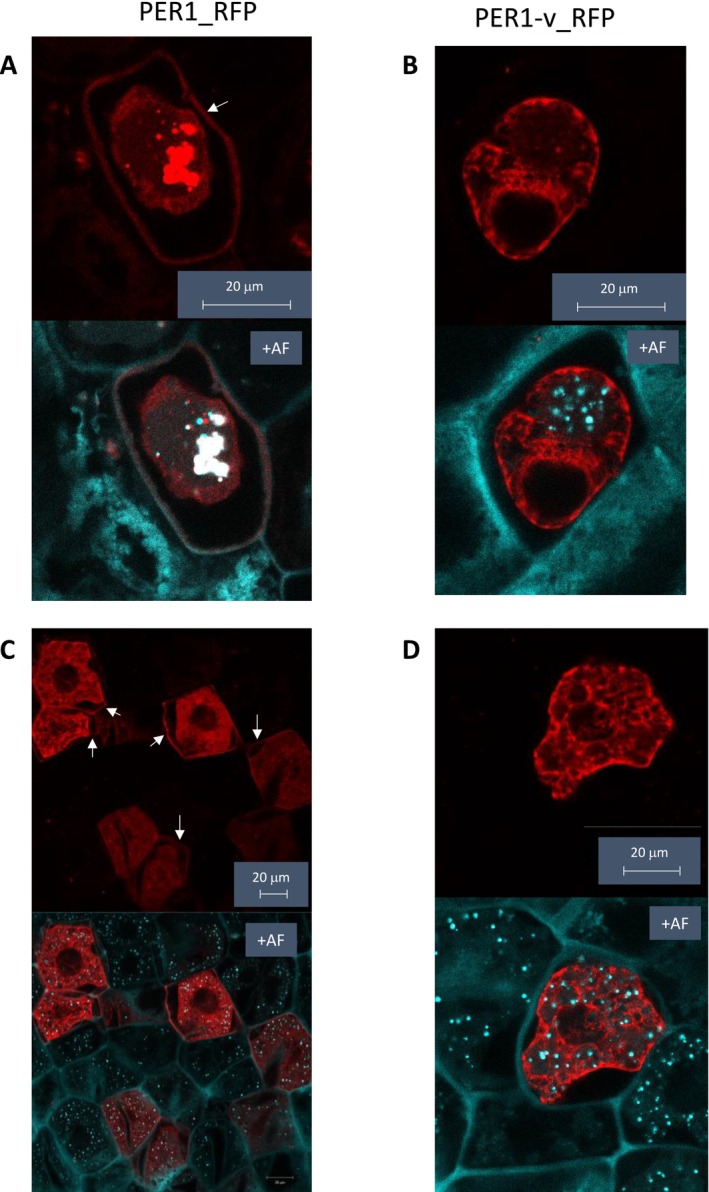
Example images of sections of plasmolysed wheat endosperm cells transiently expressing PER1_RFP and PER1‐v_RFP proteins. Pairs of images show RFP fluorescence signal alone in the top panel and combined with autofluorescence (+AF) in the lower panel to show cell walls. Plasmolysis caused complete (A, B) or partial (C, D) separation of cell contents from cell wall allowing identification of RFP signal from cell wall indicated by arrows. Cell wall localisation of RFP signal is present in cells expressing PER1_RFP (A, C) but not those expressing PER1‐v_RFP (B, D).

To investigate whether the missense SNP could explain the effect of PER1‐v allele (as opposed to the expression difference; Figure 1B), we made constructs with and without the missense SNP to encode PER1 and PER1‐v, both fused at C‐terminal to RFP reporter and driven by the endogenous promoter. We transiently transformed wheat endosperm tissue with plasmids containing these constructs. For both constructs, the RFP signal was mostly in the cytoplasm, possibly as a result of high expression overloading the capacity of the cell to normally process the protein which is predicted to require signal peptide cleavage and N‐glycosylation. However, in plasmolysed cells expressing PER1_RFP, we also observed RFP signal associated with the cell wall, whereas in plasmolysed cells expressing PER1‐v_RFP, we never observed cell wall RFP signal (examples shown in Figure 2, the complete set of images with clearly plasmolysed cells is in Supplemental Figures S1–S4). In total, we observed cell wall RFP signal in 41 of 43 plasmolysed cells with PER1_RFP, whereas there were none in 40 plasmolysed cells expressing PER1‐v_RFP. Since we would expect most or all AX ferulate dimerization to occur in the cell wall (Burr and Fry 2009), the failure of PER1‐v to localise to the cell wall means it is likely defective in function.

### 2.6 Genome‐Wide Association Validation of PER1 in Elite Germplasm

**FIGURE 3:**
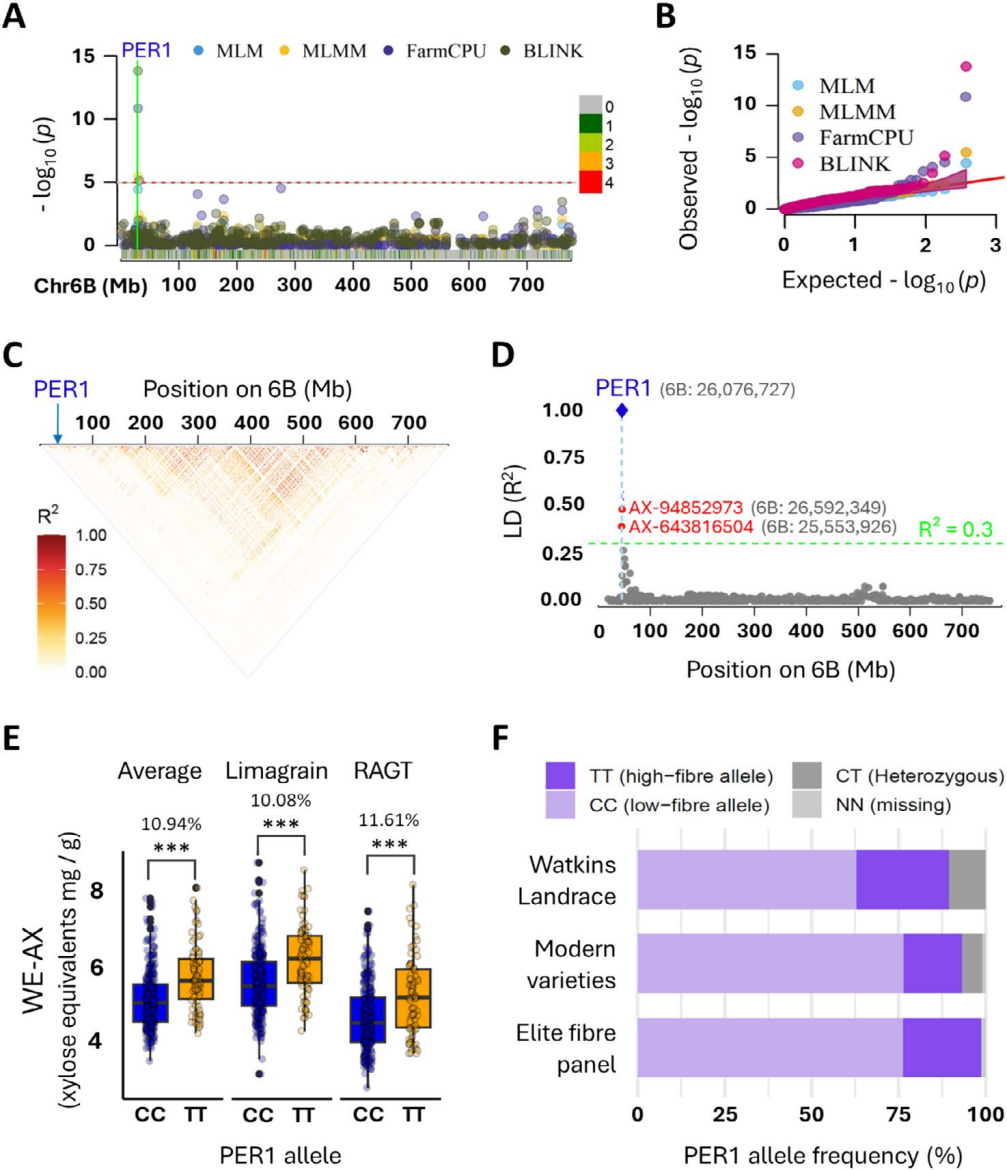
Genome‐wide association supports causal PER1 missense SNP on chromosome 6B controlling soluble fibre (WE‐AX) in wheat. (A) Manhattan plot of the multi‐model GWAS for WE‐AX across chromosome 6B. Each point is a SNP; colours denote the statistical method used. The dashed red line marks the genome‐wide significance threshold (−log_10_ (*p*) = 5). All models' peak significance is PER1 SNP. SNP density in 1 Mb bins is indicated by colour on horizontal bar at bottom. (B) Q‐Q plot demonstrating a robust SNP‐trait association across all four models. (C) Chromosome‐wide triangular linkage disequilibrium (LD) heatmap (*R* ^2^) for 6B. PER1 (arrow) lies outside any extended haplotype block; rapid LD decay explains the weak tagging by neighbouring array markers. (D) Pairwise *R* ^2^ between the PER1 missense SNP (blue diamond) and 774 Axiom markers across 6B. The nearest array SNPs (red dots) show low LD with PER1. (E) Phenotypic effect of the PER1 alleles. Boxplots show WE‐AX content in EFP lines carrying the common PER1 allele (CC, blue) and the high soluble‐fibre PER1‐v allele (TT, orange), across two commercial breeding sites (Limagrain and RAGT) and their overall average. Each point represents an individual line. In all comparisons, lines carrying the PER1‐v allele had significantly higher WE‐AX than those with the PER1 allele (*p* < 0.001). (F) Allele frequencies of the PER1 missense SNP in three different wheat genetic resources.

The effect of the single missense SNP on PER1 localisation strongly suggests that this is the cause of the 6B QTL for SDF. If this is the case, this SNP (6B: 26076727) represents a perfect marker for the trait. We carried out a genome‐wide association study on the Elite High‐Fibre Panel (EFP) of modern winter wheats; the PER1 missense SNP was the top association for WE‐AX on chromosome 6B across all four GWAS models (MLM, MLMM, FarmCPU, BLINK), with P values ranging from 1.46 × 10^−14^ to 3.57 × 10^−5^ (Figure 3A,B). The locus lies in a distal 6B region with rapid LD decay; accordingly, the PER1 marker shows no extended LD with flanking array SNPs (Figure 3C,D). This is expected when the functional polymorphism itself is genotyped: the association collapses to the causal SNP, while neighbouring markers (which often differ in minor‐allele frequency and have been uncoupled by historical recombination) show low pairwise *R* ^2^. Together, the cross‐model concordance and absence of an LD shoulder around PER1 provide strong GWAS‐based validation that the PER1 missense SNP is the causal variant underlying the 6B WE‐AX QTL in elite germplasm. Boxplots of WE‐AX content clearly show that lines carrying the PER1‐v allele (T) had higher WE‐AX by around 11% on average than those with the common allele (C) (Figure 3E).

The causal allele PER1‐v was found to be not rare in diverse wheat germplasm (Figure 3F): 22.7% in the EFP, 17.0% in modern global varieties and 26.8% in Watkins landraces. These values suggest that PER1‐v is not deleterious and has been maintained in elite breeding pools without apparent yield penalty, supporting its potential for breeding wheat with increased soluble dietary fibre.

## 3 Discussion

Soluble dietary fibre such as WE‐AX may confer health benefits via several mechanisms including fermentation to release short‐chain fatty acids in the colon, and by increasing viscosity which may reduce the rate of glucose release from starch in the duodenum (Gill et al. 2021). Extractability of AX in water is determined by several structural factors of which cross‐linking via ferulate dimerization is likely the most important (Saulnier et al. 2007). The AX in the starchy endosperm, and particularly the WE‐AX fraction, has less ferulate [AX ~0.5%; WE‐AX ~0.1% (Freeman et al. 2017)], and therefore, potential for cross‐linking than AX from other tissues including the bran which has high AX content that is largely non‐extractable in water (Gebruers et al. 2008).

It has been shown that oxidative coupling of ferulate that results in dimers and higher oligomers bound to AX in grass cell walls occurs in the apoplast and is mediated by peroxidases (Burr and Fry 2009) but these have not been identified. Here, we show that the dimerization of ferulate on wheat endosperm AX is mediated by the PER1 protein, since both a natural allele and an induced KO mutant result in lower dimerization (Tables 2, 3, 4). The effect of the natural allele could conceivably be from changes in expression of PER1, PER1&2 and PER2 associated with the Valoris 6B genotype (Figure 1) due to *cis* factors (although PER2 expression is actually markedly increased). However, our finding that a single amino acid residue change prevents localisation of PER1‐v to the cell wall (Figure 2), the likely site for AX dimerization, identifies this as the more probable cause. The same change would be present in the PER1&2 protein, so it seems likely this form would be similarly affected (although this was not tested). We therefore attribute the 6B QTL effect on SDF to this SNP resulting in a mislocalised PER1 that lowers the capacity for AX ferulate dimerization, resulting in a higher proportion of AX being extractable in water.

PER1 is a member of a large gene family of class III haem peroxidases that are specific to green plants. These peroxidases apparently all catalyse the same reaction in vitro, generating radical oxygen species from H_2_O_2_ and it has been hypothesised that in vivo specificity is achieved by localisation close to the intended substrate (Francoz et al. 2015). This is supported by observation of precise localisation of peroxidases associated with lignification in Arabidopsis (Hoffmann et al. 2020) and has been demonstrated for a peroxidase that binds to a particular cell wall microdomain on the polysaccharide homogalacturonan to function in mucilage extrusion (Francoz et al. 2019) and for a lignin peroxidase that localises to the Casparian strip via binding to another protein (Lee et al. 2013). It is therefore probable that a large family of peroxidase genes is required for the multiplicity of peroxidase binding sites that confer specificity of function. This is consistent with our result showing that PER1 is present in the cell wall whilst PER1‐v is not, and this difference is associated with decreased dimerization of AX ferulate. The single amino acid difference between the two forms of the peroxidase is at a serine residue in PER1 which is predicted by AlphaFold to be involved in H‐bonding in an α‐helix on the exterior of the protein (Figure S5). This is changed in PER1‐v to the aromatic amino acid phenylalanine which would prevent H‐bonding and disrupt the α‐helix. PER1 and PER2 are in a subclade of Group VI of the class III peroxidase gene family as defined in (Passardi et al. 2004) (Figure S6). PER1 and PER2 are only expressed in grain out of the major wheat tissues (Figure S6) suggesting they may have specific properties related to the unusual structure of grain AX. Apart from the practical application to wheat breeding, the identification of PER1 also represents the first peroxidase with strong evidence of a role in cross‐linking AX chains, an important step forward in our broader understanding of grass cell walls.

The identification of PER1 as the cause of variation in SDF is supported by our GWAS results which provide strong population‐level validation (Figure 3). The absence of an LD shoulder around PER1, combined with highly significant single‐marker associations, is precisely the signature expected when the causal variant is directly assayed. The presence of the PER1‐v allele in 23% of the EFP and 17% of global modern cultivars compared to 27% in Watkins suggests that the trait does not adversely affect yield or quality and hence the diagnostic marker could be used to not only select for high WE‐AX in flour in breeding programmes but identify current cultivars which could be specified by bakers and food processors to increase the fibre content of products today. Furthermore, higher contents of WE‐AX would also be present in whole grain products as most of the WE‐AX in whole grains is present in the white flour (endosperm) as opposed to the bran fraction (Gebruers et al. 2008; Lovegrove et al. 2020). Since we elucidated the mode of action of the PER1‐v allele and tied it mechanistically to a specific SNP that induces the mislocalisation, we can confidently predict that the marker will always identify the higher WE‐AX trait in any wheat germplasm (a ‘perfect marker’). Understanding of mechanism will also help us to predict how it may behave in combination with other alleles affecting wheat DF as knowledge increases.

Our work shows a clear route to healthier wheat‐based foods by using the PER1‐v marker to select wheat varieties for processing and breed new ones as outlined above that could be carried out now. For example, in the UK, the wide consumption of bread across socio‐economic groups (Lockyer and Spiro 2020) means that deploying the PER1‐v allele to all breadmaking wheats would result in wide benefits. The use of PER1‐v would increase SDF intake, which may have particular benefits due to its viscosity and fermentability (Gill et al. 2021), and could contribute to a wider effort to increase dietary fibre intake, thereby reducing the risk of chronic diseases associated with the Western diet (Reynolds et al. 2019).

## 4 Methods

### 4.1 Biparental Population

We grew the Yumai34 × Valoris population in the field in 2018 and in the glasshouse in 2022 in randomised designs at Rothamsted Research, UK. In both experiments, we selected DHLs as having the same genotype for all Axiom genotyping markers on 35 K SNP platform within the 1A, 1B, 6B QTLs and assigned these to 4 pools of contrasting parental genotype at 1B and 6B but all with Yumai34 genotype at 1A. Three biological reps of these pools from different blocks were used from the 2018 field experiment for transcriptome analysis and from the 2022 glasshouse experiment for ferulate determination.

### 4.2 RNA‐Seq Analysis

Total RNA was isolated from developing endosperm of 20 selected DHLs at 17 days post anthesis as previously described (Pellny et al. 2012, 2020) to give 3 biological replicates of each pool. Library preparation and mRNA sequencing were done by Novogene (HK, China). We mapped reads to IWGSC refseq 1.1 genome using HISAT2 (Kim et al. 2015), visualised alignments using Integrative Genomics Viewer (Robinson et al. 2011) and estimated transcript abundance as normalised counts using DESeq2 (Love et al. 2014). Normalised counts for TraesCS6B02G042500.1 and TraesCS6B02G042600.1 gene models in reference were reallocated to PER1, PER1&2 and PER2 based on manually determined ratios of counts of read pairs that could be clearly assigned to these.

### 4.3 PER1 Knock‐Out Mutant Lines

From the database of mutations in the exome sequenced collection of mutagenised wheat cv Cadenza available at Ensembl Plants, we identified line Cadenza1644 carrying a G‐>A mutation at 26076941 on chr6B causing a premature stop codon Trp94STOP in exon 2 of PER1. We backcrossed this line twice to Cadenza, allowed to self‐fertilise over two further generations and identified BC2F2 homozygotes for both mutation and wild‐type allele using crossing and genotyping procedures previously described (Pellny et al. 2020); subgenome‐specific primers for genotyping are given in Table S2. We grew these lines in randomised designs in two glasshouse experiments with independent lines acting as replicates per genotype, first on BC2F2 generation (*n* = 7) then on BC2F3 generation (*n* = 5,6).

### 4.4 Endosperm (White Flour) Fraction

White flour was produced using a Micro Scale Labmill FQC‐2000 (METEFÉM SZÖVETKEZET, Hungary, 1047 Budapest, TINÓDI U. 28–30.), following conditioning of mature grain to 15.5% moisture content for 3 h at room temperature on Spiramix (Denley). Initial moisture content of grain was determined using Bruker Minispec mq‐20 NMR analyser using an in‐house developed calibration. All biochemical analyses were carried out on the white flour fraction ≤ 150 μm.

### 4.5 WE‐AX Content Determinations

WE‐AX content was determined in the Cadenza mutant experiment by analysis of water‐extractable monosaccharides from 5 mg of white flour as described in Corado et al. (Corrado et al. 2020). WE‐AX content in samples from the EFP panel for GWAS was determined by the efficient pentosan method (Hernández‐Espinosa et al. 2024).

### 4.6 Ferulate Monomer and Dimer Analysis

We analysed the bound ferulate monomer and dimer content of total and water‐extractable endosperm fractions from mature grain by HPLC analysis as previously described (Freeman et al. 2017) using pure ferulate dimer standards kindly supplied by Professor John Ralph (Lu et al. 2012).

### 4.7 Transient Expression of PER1_RFP and PER1‐v_RFP in Immature Wheat Endosperm

We designed a version of PER1 CDS (TraesCS6B02G042500.1) that contained additional cloning sites immediately before the natural NcoI site present over the ATG codon and an HpaI site at the C terminal end of the gene that involved the addition of an extra serine residue to the protein. Nine synonymous changes were also engineered into the coding sequence to facilitate cloning while retaining the natural PER1 protein sequence. We designed PER1‐v from this with a single bp change corresponding to the Valoris allele missense SNP giving rise to the Ser51Phe peptide sequence change. From alignment analysis of the 5′ upstream sequences of PER1 and the Chinese Spring, A, and D homeologues and other wheat relatives, we selected 2135 bp of the PER1 sequence as likely to contain a complete promoter including the 5′ transcribed untranslated sequences. Bases immediately upstream of the ATG codon were modified to contain the same restriction sites as engineered into the PER1 and PER1‐v gene sequences for cloning purposes and to remove the out‐of‐frame ATG codon present 5 bp upstream of the PER1 start codon. The designed PER1, PER1‐v and PER1 promoter sequences were synthesised by Genscript (Oxford, UK) and transferred using standard cloning techniques to pRRes208, a biolistic vector backbone. Two plasmids were generated with the PER1 promoter driving either PER1 [pRRes208.618] or PER1‐v [pRRes208.617]. In each construct, the PER1 sequences were fused in frame upstream of a six amino acid linker sequence and the fluorescence marker gene Tag‐RFP_T. Immature wheat endosperm sections were isolated from 7 to 10 dpa wheat seeds cv Cadenza, as described in (Jones et al. 2008). The tissues were bombarded on the same day as isolation using 0.6 μm gold particles (Bio‐Rad Laboratories Ltd., UK) coated with the constructs pRRes208.617, pRRes208.618 or pRRes.380, a plasmid containing Tag‐RFP under the control of the constitutive rice actin promoter which acted as a positive control. Bombardment was carried out using the PDS‐1000/He particle gun with 650 psi rupture pressure and 29″ Hg vacuum; full details of bombardment parameters can be found in (Sparks and Doherty 2020). Plates were incubated in the dark at 22°C for 1 day prior to bioimaging.

### 4.8 Imaging of Wheat Endosperm Transiently Expressing PER1_RFP and PER1‐v_RFP

Transformed, immature wheat endosperm sections were mounted on glass slides with coverslips for imaging with a Zeiss LSM 780 confocal microscope (Carl Zeiss Ltd. Cambourne, Cambridge, UK). The endosperm tissue was mounted in distilled water for initial imaging and the water replaced under the coverslip with 2 M NaCl by capillary action to induce plasmolysis. Cells were monitored for up to 10 min to observe plasmolysis and imaged with the 40× objective using 405 nm excitation, 428–490 nm emission for cell wall auto‐fluorescence and 561 nm excitation, 580–700 nm emission for Tag‐RFP.

### 4.9 KASP Marker for Valoris Missense SNP

We used a KASP marker designed to Valoris chr6B 26 076 727 SNP to confirm SNP genotype of the selected lines in Yumai34 × Valoris population and in elite high fibre panel. Primers and PCR conditions are given in Table S2.

### 4.10 Genome‐Wide Association Study and Allele Frequency Analysis

We carried out a genome‐wide association study (GWAS) using an independent panel of 384 elite UK wheat breeding lines (Elite Fibre Panel, EFP). Lines were grown in two different commercial breeding sites (Limagrain and RAGT) and were genotyped with the Axiom TaNG SNP array and phenotyped for WE‐AX content using the pentosan method. Association mapping was performed on the WE‐AX averages using four GWAS models (MLM, MLMM, FarmCPU, BLINK). Allele frequency of the PER1 SNP was determined in the EFP, a panel of modern global varieties, and the Watkins landrace collection (Cheng et al. 2024). Pairwise linkage disequilibrium (LD, *R* ^2^) between the PER1 SNP and neighbouring markers across chromosome 6B was calculated to assess haplotype structure.

## Author Contributions

Wheat DF project initiation: P.R.S., A.L., S.G. Experiment planning: R.A.C.M., P.R.S., S.G. and A.L. Experimental – RNAseq: T.K.P.; genotyping and crossing of mutants: J.F., A.P., J.B.; monosaccharide: O.K.; ferulate: O.K., M.O.; RFP fusion constructs: A.H.; endosperm transformation: C.A.S.; bioimaging: K.H.; genotyping PER1‐v SNP: M.L.‐W.; GWAS phenotyping: A.P. GWAS analysis: A.K.A. Other bioinformatic and data analyses: R.A.C.M. Paper writing: R.A.C.M. with input from other authors.

## Funding

This work was funded by the Biotechnology and Biological Sciences Research Council (BBSRC) of UK grants BB/P016855/1, BB/T013923/1 and BB/X011003/1 Institute Strategic Programme delivering Sustainable Wheat.

## Conflicts of Interest

The authors declare no conflicts of interest.

## Supporting information


**Table S1:** Amounts of ferulate monomer and dimers in total endosperm of Cadenza BC2F2 lines segregating for KO mutation in PER1.
**Table S2:** PCR conditions and primers.
**Figures S1–S4:** Further images from endosperm grain sections transiently expressing PER1_RFP and PER1‐v_RFP, where clear cell plasmolysis was observed.
**Figure S5:** AlphaFold predicted structure of PER1 protein with Ser51 residue that is changed in PER1‐v highlighted.
**Figure S6:** Phylogeny of subclade within class III peroxidase family containing PER1 and PER2 genes with expression abundance for wheat genes.

## Data Availability

All raw data including RNAseq reads are available from the authors on request.
