# Supplementary material for: A High Soluble‐Fibre Allele in Wheat Encodes a Defective Cell Wall Peroxidase Responsible for Dimerization of Ferulate Moieties on Arabinoxylan
Source: Plant Biotechnol J. 2026 Jan 3;24(5):2751–61. doi: 10.1111/pbi.70527 (PMC13110136; doi:10.1111/pbi.70527)
Supplement: Supplementary file 1 — Table S1: Amounts of ferulate monomer and dimers in total endosperm of Cadenza BC2F2 lines segregating for KO mutation in PER1. Table S2: PCR conditions and primers. Figures S1–S4: Further images from endosperm grain sections transiently expressing PER1_RFP and PER1‐v_RFP, where clear cell plasmolysis was observed. Figure S5: AlphaFold predicted structure of PER1 protein with Ser51 residue that is changed in PER1‐v highlighted. Figure S6: Phylogeny of subclade within class III peroxidase family containing PER1 and PER2 genes with expression abundance for wheat genes. [file PBI-24-2751-s001.docx]

**Supporting Information for “A defective peroxidase allele causes high soluble fibre in wheat, providing a route to healthier wheat-based foods”**

Table S1. Amounts of ferulate monomer (FA) and dimers (diFA) in total endosperm of Cadenza BC2F2 lines segregating for KO mutation in PER1. Results from a 1-way ANOVA of mutation showing means for each group (n=7) and F probability for effect.

|  |  |  | diFA / FA (w/w) | | | | | |  |
| --- | --- | --- | --- | --- | --- | --- | --- | --- | --- |
| means |  | Dimer-isation [tot diFA / (tot diFA + FA)] | diF8-8AT | diF8-8 | diF8-5 | diF5-5 | diF8-0-4 | diF8-5BF | FA (μg / g dwt) |
|  | mutant | 15.5% | 0.018 | 0.036 | 0.049 | 0.027 | 0.052 | 0.053 | 88.8 |
|  | null | 17.0% | 0.024 | 0.04 | 0.05 | 0.035 | 0.056 | 0.061 | 79.3 |
| F prob. |  |  |  |  |  |  |  |  |  |
|  | mutation | 0.037 | 0.019 | 0.227 | 0.830 | 0.030 | 0.346 | 0.046 | 0.194 |

| role | PCR conditions | product size | primer type | primer name | primer seq |
| --- | --- | --- | --- | --- | --- |
| genotyping of premature stop codon mutation from Cadenza1644 by Sanger sequencing |  | 318 | forward | prTYQ39_Per1B1644F | ATGCAAGACTGACAGACCGA |
|  |  |  | reverse | prTYQ40_Per1B1644R | CGAATGCGTGTCAATTGTCA |
| KASP codominant marker for Valoris missense SNP in PER1 | 65C – 57C hotstart for 10 cycles, followed by 45 cycles of 94C and 57 C | 47 | ref_allele | PER1_S1_Fam | GAAGGTGACCAAGTTCATGCTTGACCATGGACTGCTTCACGG |
|  |  |  | Valoris_allele | PER1_S1_Vic | GAAGGTCGGAGTCAACGGATTCTTGACCATGGACTGCTTCACGA |
|  |  |  | common | PER1_S1_Com | ACCTGGAGGGCATTGTCCGGTA |

Table S2. PCR conditions and primers.


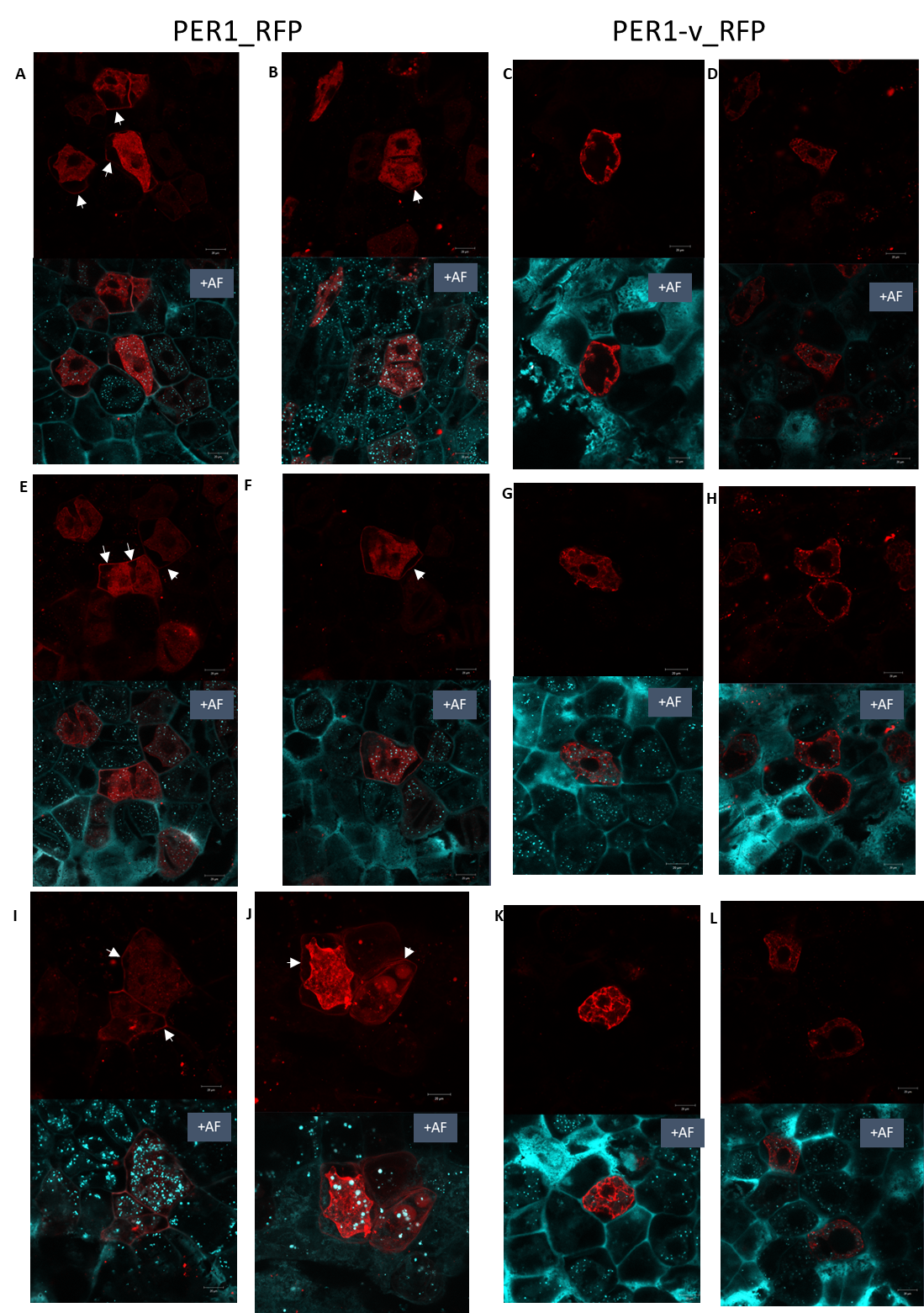


Figures S1. Images from endosperm grain sections transiently expressing PER1_RFP and PER1-v_RFP where clear cell plasmolysis was observed. Pairs of images show RFP fluorescence signal alone in top panel and combined with autofluorescence (+AF) in lower panel to show cell walls. Plasmolysis separation of cell contents from cell wall allowing identification of RFP signal from cell wall indicated by arrows. Cell wall localisation of RFP signal is present in cells expressing PER1_RFP (**A**, **B, E, F, I, J**) but not those expressing PER1-v_RFP (**C**, **D, G, H, K, L**).


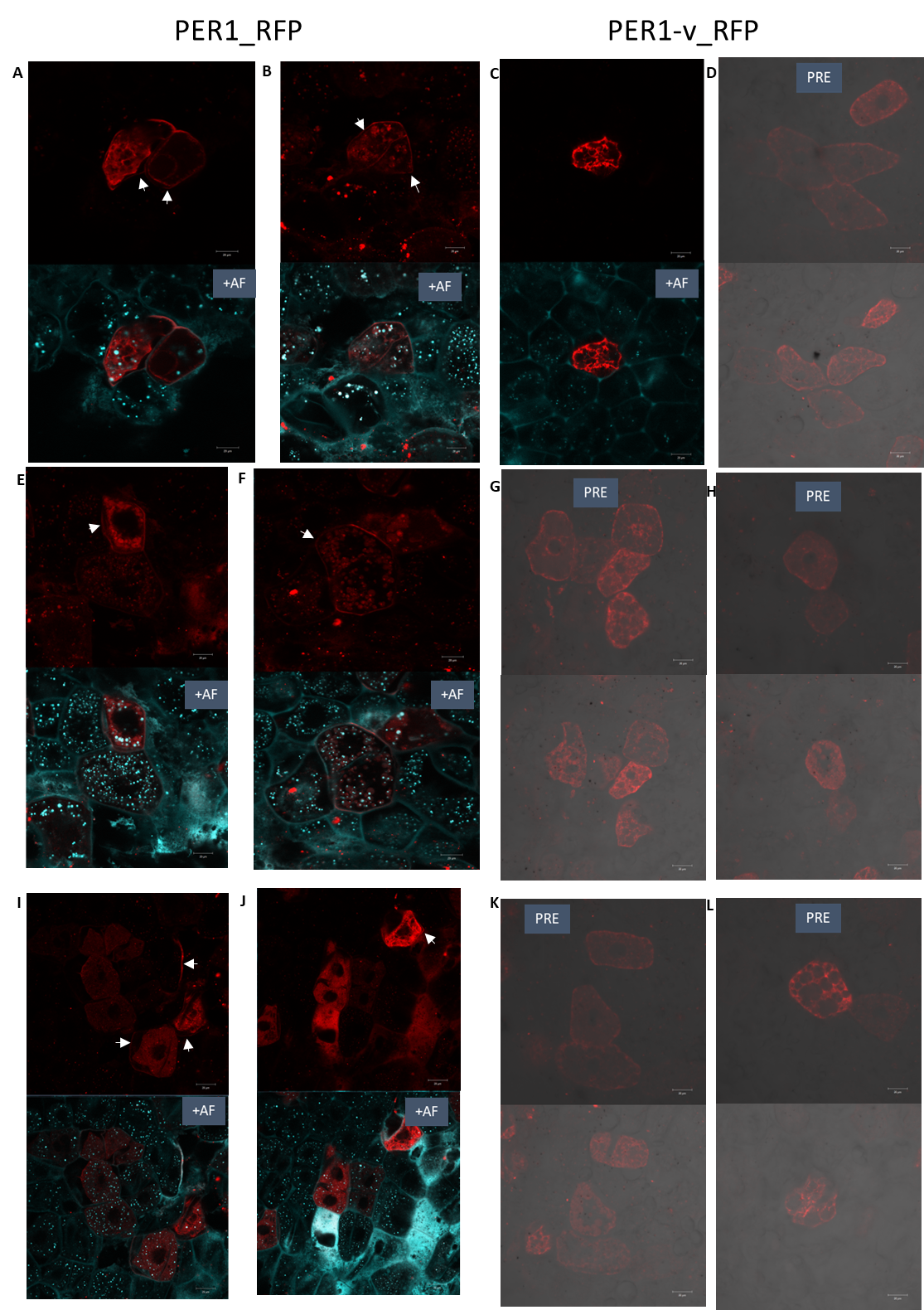


Figures S2. Further images from endosperm grain sections transiently expressing PER1_RFP and PER1-v_RFP where clear cell plasmolysis was observed. Details as for Fig. S1, except for image pairs (**D, G, H, K, L)** where image in upper panel is pre-plasmolysis and after plasmolysis in lower panel.


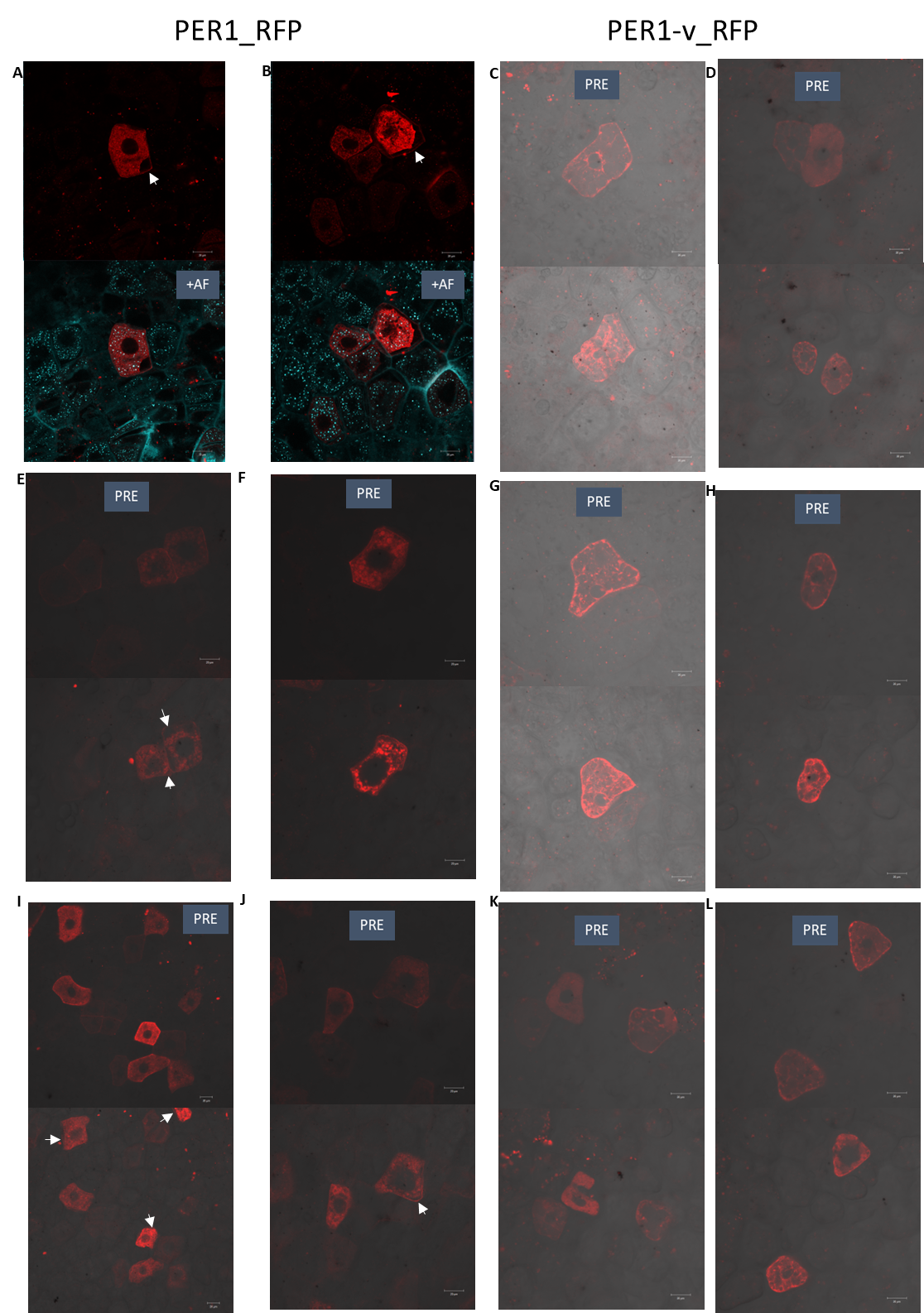


Figures S3. Further images from endosperm grain sections transiently expressing PER1_RFP and PER1-v_RFP where clear cell plasmolysis was observed. Details as for Fig. S1, except for image pairs (**C-L)** where image in upper panel is pre-plasmolysis and after plasmolysis in lower panel. Image pair **F** is the single exception where we could not see clear cell wall localisation of RFP signal in cells expressing PER1_RFP.


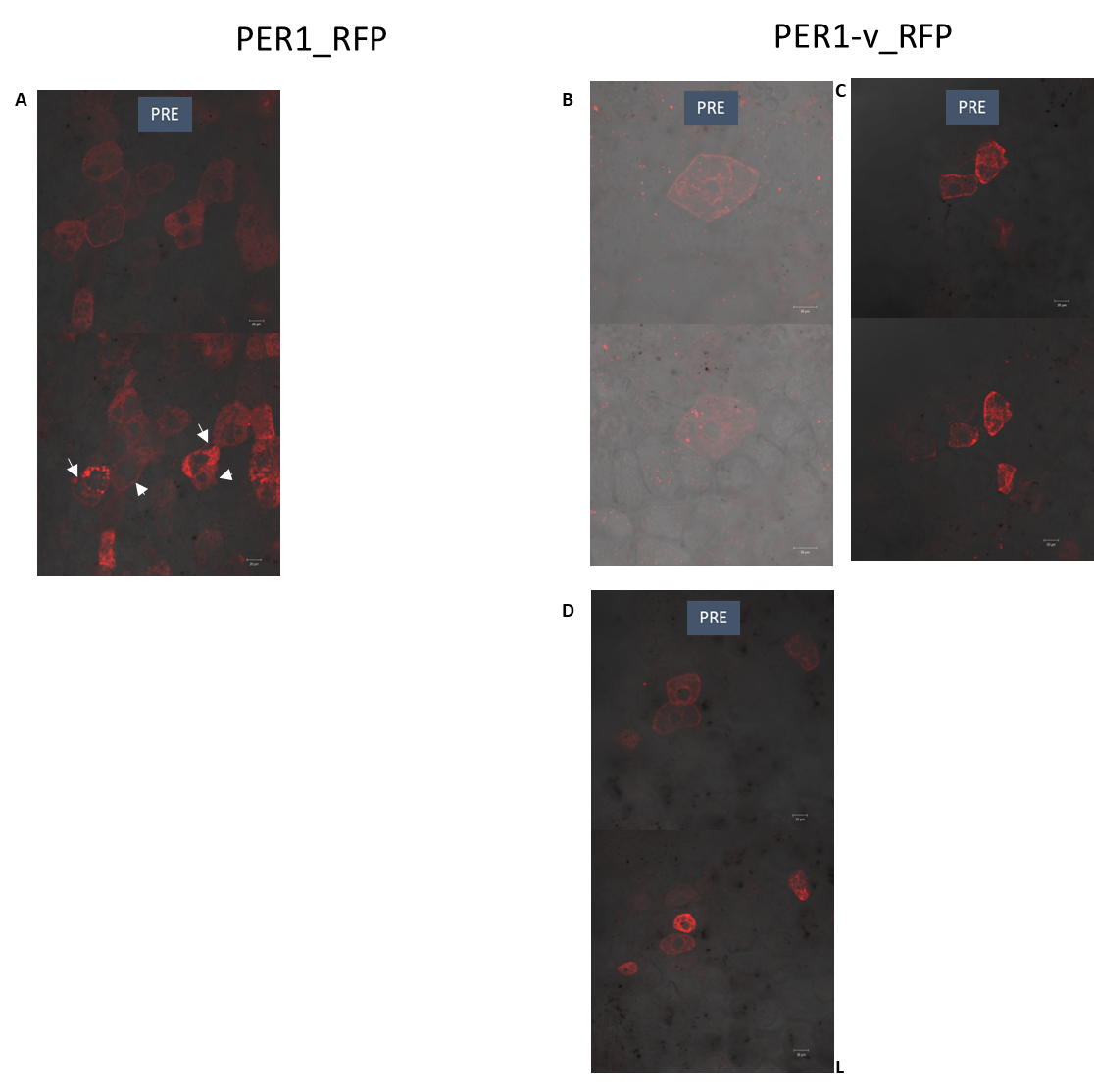
Figures S4. Further images from endosperm grain sections transiently expressing PER1_RFP and PER1-v_RFP where clear cell plasmolysis was observed. Pairs of images show pre-plasmolysis image in upper panel and after plasmolysis in lower panel. Plasmolysis causes separation of cell contents from cell wall allowing identification of RFP signal from cell wall indicated by arrows. Cell wall localisation of RFP signal is present in cells expressing PER1_RFP (**A**) but not those expressing PER1-v_RFP (B, **C**, **D**).


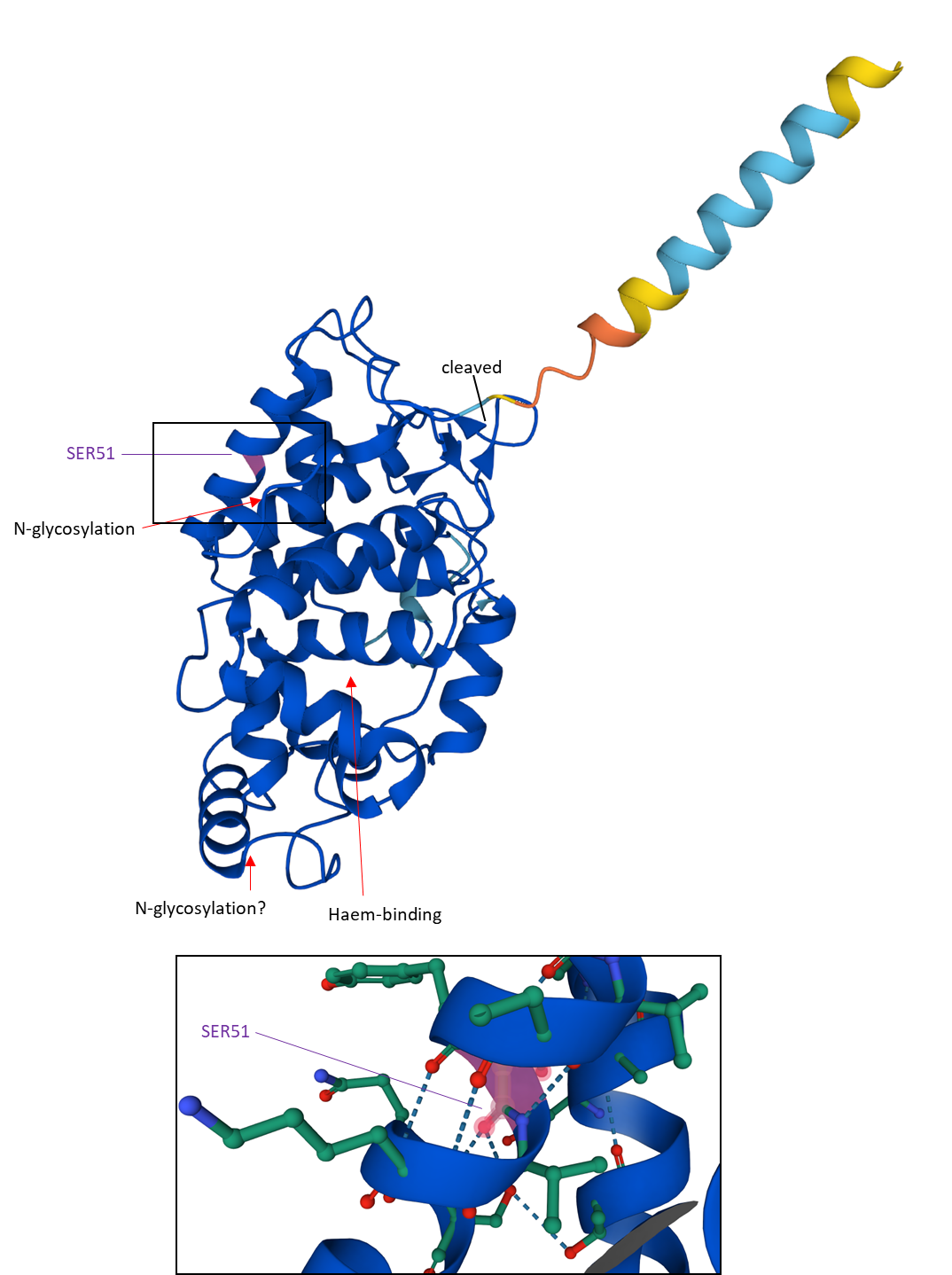


Figure S5. AlphaFold predicted structure of PER1 (TraesCS6B02G04600.1) protein with Ser51 residue that is changed in PER1-v highlighted. Predicted N-glycosylation Asn, haem-binding and signal peptide cleavage sites are indicated.


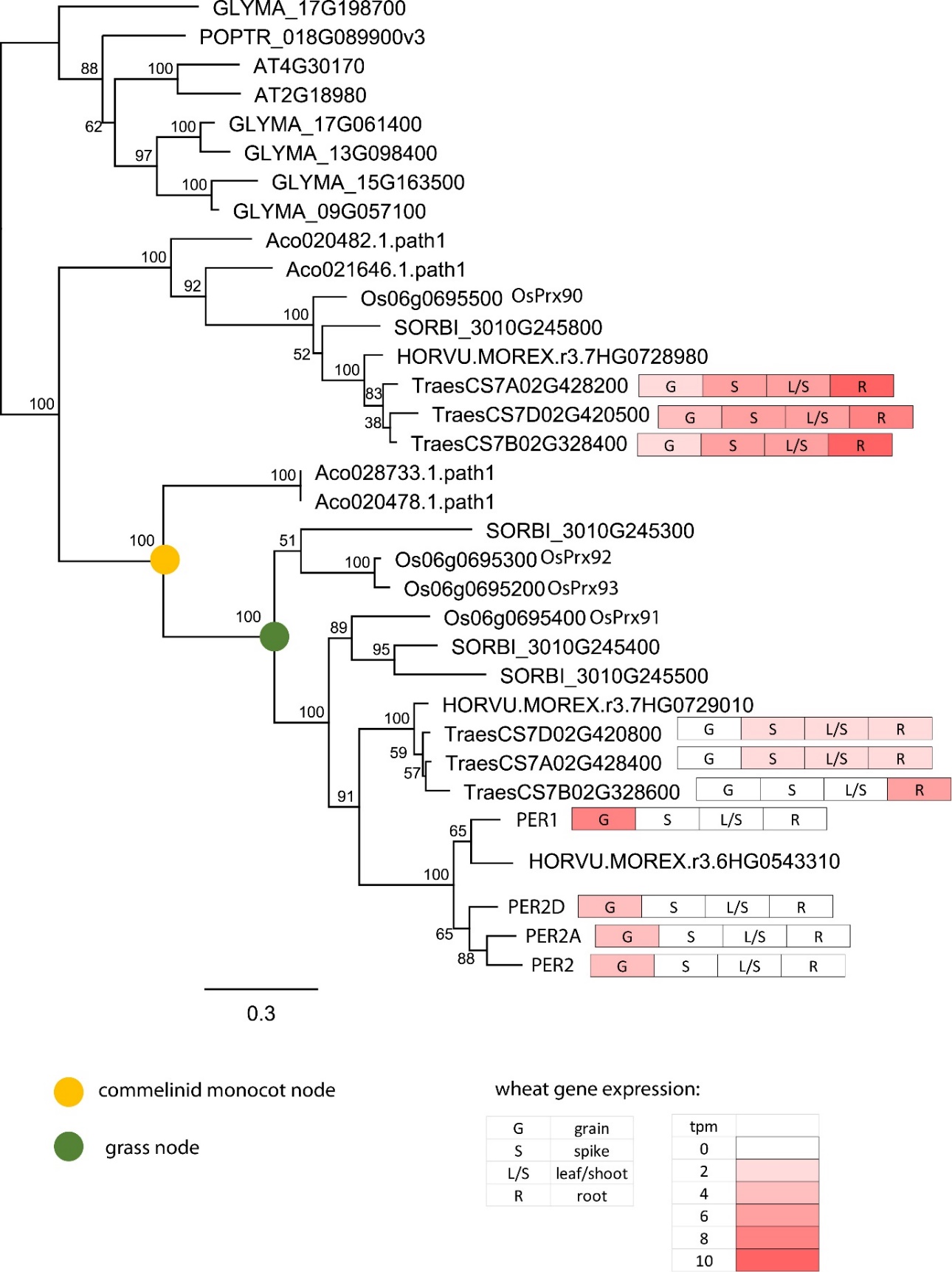


Figure S6. Phylogeny of subclade within class III peroxidase family containing PER1 and PER2 genes. For phylogenetic analyses of protein sequences, we selected the rice ortholog of PER genes (OsPRX91) and its three closest paralogs (OsPrx90, OsPrx92, OsPrx93) which are all neighbouring genes on rice chromosome 6. We then selected all orthologs of these from wheat, Arabidopsis, *Ananas comosus*, *Glycine max*, *Hordeum vulgare*, *Populus trichocarpa* and *Sorghum bicolor* as defined Ensembl Plants release 56 (Yates et al., 2021) and aligned with MUSCLE (Edgar, 2004) and generated phylogenetic tree with Phyml (Guindon et al., 2010) as previously described (Pellny et al., 2012). Wheat expression data was taken from wheat-expression.com (Borrill et al., 2016) averaged across 34 studies encompassing many genotypes and environments.
